# Fangchinoline alleviates cognitive impairments through enhancing autophagy and mitigating oxidative stress in Alzheimer’s disease models

**DOI:** 10.3389/fcell.2023.1288506

**Published:** 2023-12-11

**Authors:** Lilin Yi, Man Luo, Maoju Wang, Zhifang Dong, Yehong Du

**Affiliations:** ^1^ Ministry of Education Key Laboratory of Child Development and Disorders, National Clinical Research Center for Child Health and Disorders, Chongqing Key Laboratory of Translational Medical Research in Cognitive Development and Learning and Memory Disorders, Pediatric Research Institute, Children’s Hospital of Chongqing Medical University, Chongqing, China; ^2^ Institute for Brain Science and Disease of Chongqing Medical University, Chongqing, China

**Keywords:** alzheimer’s disease, amyloid-β, BACE1, autophagy, oxidative stress, cognition

## Abstract

**Introduction:** Alzheimer’s disease (AD) is a debilitating, progressive, neurodegenerative disorder characterized by the deposition of amyloid-β (Aβ) peptides and subsequent oxidative stress, resulting in a cascade of cytotoxic effects. Fangchinoline (Fan), a bisbenzylisoquinoline alkaloid isolated from traditional Chinese herb Stephania tetrandra S. Moorec, has been reported to possess multiple potent biological activities, including anti-inflammatory and antioxidant properties. However, the potential neuroprotective efficacy of Fan against AD remains unknown.

**Methods:** N2A^APP^ cells, the mouse neuroblastoma N2A cells stably transfected with human Swedish mutant APP695, were served as an *in vitro* AD model. A mouse model of AD was constructed by microinjection of Aβ_1–42_ peptides into lateral ventricle of WT mice. The neuroprotective effects of Fan on AD were investigated through a combination of Western blot analysis, immunoprecipitation and behavioral assessments.

**Results and discussion:** It was found that Fan effectively attenuated the amyloidogenic processing of APP by augmenting autophagy and subsequently fostering lysosomal degradation of BACE1 in N2A^APP^ cells, as reflected by the decrease in P62 levels, concomitant with the increase in Beclin-1 and LC3-II levels. More importantly, Fan significantly ameliorated cognitive impairment in an Aβ_1–42_-induced mouse model of AD via the induction of autophagy and the inhibition of oxidative stress, as evidenced by an increase in antioxidants including glutathione reductase (GR), total antioxidant capacity (T-AOC), nuclear factor erythroid-2-related factor 2 (Nrf2), heme oxygenase-1 (HO-1), and superoxide dismutase-1 (SOD-1) and a decrease in pro-oxidants including hydrogen peroxide (H_2_O_2_) and inducible nitric oxide synthase (i-NOS), coupled with a reduction in apoptosis marker, cleaved caspase-3. Taken together, our study demonstrate that Fan ameliorates cognitive dysfunction through promoting autophagy and mitigating oxidative stress, making it a potential therapeutic agent for AD.

## Introduction

Alzheimer’s disease (AD) is the most common neurodegenerative disease in the elderly, affecting exceeding 50 million individuals globally (Hodson, 2018; GBD 2019 Dementia Forecasting Collaborators, 2022). The main pathological hallmark of AD is the extracellular senile plaques, formed by the deposition of amyloid-β (Aβ) peptides, which derive from the sequential enzymatic cleavage of amyloid precursor protein (APP) by the β-secretase and γ-secretase (Bossy-Wetzel et al., 2004; Baranello et al., 2015). β-site APP-cleaving enzyme 1 (BACE1) directly cleaves APP to generate transmembrane C-terminal fragments (β-CTFs) of either 89 or 99 residues (C89 or C99), which are subsequently cleaved by γ-secretase to produce Aβ peptides (Estus et al., 1992; Golde et al., 1992; Vassar et al., 1999; Cai et al., 2001). Serving as the primordial and rate-determining enzyme in the generation of Aβ (Willem et al., 2009), and given its increased expression in the brains of elderly individuals and AD patients (Yang et al., 2003; Fukumoto et al., 2004), BACE1 has become a pivotal target for the prevention and treatment of AD. An expanding array of research supports the efficacy of both genetic knockdown of BACE1 and the use of BACE1 inhibitors in reducing Aβ levels, leading to improvements in synaptic and cognitive dysfunction in AD model mice (Ohno et al., 2004; McConlogue et al., 2007; Neumann et al., 2015). Unfortunately, despite these promising findings, the multifaceted physiological functions of BACE1 have complicated the translation of these findings into effective treatments for AD patients. Exploration of alternative ways to inhibit BACE1 may be a more feasible avenue of investigation.

Growing body of evidence indicates that the degradation of BACE1 is mediated by two key pathways: the ubiquitin-proteasome pathway and the autophagy-lysosome pathway (Qing et al., 2004; Koh et al., 2005; Feng et al., 2017). The lysine residues at positions 203 and 382 of BACE1 have been identified as pivotal for its degradation via the ubiquitin-proteasome system (Wang et al., 2012). Concurrently, SUMOylation of BACE1 enhances its enzymatic stability and amplifies its protease activity, thereby accelerating AD progression (Bao et al., 2018). The ubiquitin-specific peptidase 8, an endosome-associated deubiquitinating enzyme, not only regulates BACE1 ubiquitination, but also promotes BACE1 accumulation in early endosomes and lysosomes (Yeates and Tesco, 2016). Meanwhile, ubiquitination at the site Lys-501 of BACE1 appears to steer it to late endosomes or lysosomes for degradation (Kang et al., 2012). It has been well documented that Lys-48-linked ubiquitin chains predominantly mediate the proteasomal degradation. Conversely, Lys-63-linked ubiquitination serves as specific signal for autophagy lysosomal degradation (Lauwers et al., 2010). Notably, BACE1 undergoes predominantly mono-ubiquitination and Lys-63-linked poly-ubiquitination (Kang et al., 2010), suggesting the pivotal role of autophagy-lysosome pathway in BACE1 degradation.

Fangchinoline (Fan), an alkaloid isolated from traditional Chinese medicine Stephania tetrandra S. Moore (Chan et al., 2021), has been reported for its potent antioxidant properties. Studies indicate that Fan exerts anti-oxidative capabilities in rats subjected to an experimental rheumatoid model, as demonstrated by the elevation in antioxidants and the reduction in pro-oxidants and reactive oxygen species (ROS) (Shan et al., 2019). Furthermore, Fan mitigates nephron damage by inhibiting p38 MAPK pathway, consequently reducing oxidative stress and inflammation in rats with streptozotocin (STZ)-induced diabetic nephropathy (Jiang Y. et al., 2020). Recent studies also find that Fan triggers autophagic function, including mTOR pathway inhibition and an increased LC3-II/LC3-I ratio, as well as decreased P62 levels in bladder cancer cells (Fan et al., 2017). In colorectal cancer cell lines, Fan has been observed to initiate autophagy via the activation of AMPK/mTOR/ULK1 signaling pathway, thereby enhancing autophagosome formation (Xiang et al., 2021). However, the effects of Fan on AD remain to be elucidated. In this study, we detected the neuroprotective effects of Fan on AD through both *in vitro* and *in vivo* assessments.

## Materials and methods

### Animals

Two-month-old male C57BL/6 mice were purchased from Vital River Laboratory Animal Technology (Peking, China). The mice were raised in a temperature and humidity controlled specific pathogen-free (SPF) room under a 12 h light and 12 h dark cycle (lights on from 7:00 a.m. to 7:00 p.m.) with free access to food and water. All animal experiments were performed in accordance with the Chongqing Science and Technology Commission guidelines and approved by the Animal Ethics Committee of Children’s Hospital of Chongqing Medical University (Approval No.: CHCMU-IACUC20220323012).

### Drugs preparation and administration

Aβ_1-42_ peptides and the reagents required for its preparation including hexafluoroisopropanol (HFIP) and dimethyl sulfoxide (DMSO) were purchased from Sigma (St. Louis, MO, USA). Aβ_1-42_ peptides were prepared as previously described (Chen et al., 2017). Briefly, 0.1 mg peptides were dissolved in 1 mL HFIP and then evaporated in a fume hood to remove the HFIP. Next, the peptides were dissolved to 5 mM with DMSO, which was then diluted to a final concentration of 100 µM with phosphate buffer saline (PBS) followed by incubation at 4°C for 48 h to form oligomers.

Mice were anesthetized with 60 mg/kg sodium pentobarbital (i.p.) and then mounted on a stereotaxic device (Stoelting, USA). The hair on the top of the mouse’s head was removed and the scalp was fully exposed. Next, the scalp was disinfected with iodophor and cut with a scalpel along the center line to fully expose the skull. After the coordinates were determined, the Aβ_1-42_ oligomers (100 μM, 2.5 μL/mouse) or PBS were microinjected into the right lateral ventricle of mouse with a drilled hole (−0.5 mm posterior, +1.1 mm lateral and −3 mm ventral relative to bregma). The needle was kept for 5 min and then removed slowly after injection to make the Aβ_1-42_ oligomers fully spread. Then, the scalp was sutured and the mice were placed on a 37°C electric heating pad to maintain their body temperature. The mice were finally put back into their cages when they were awake.

Fan was purchased from Chenguang Biology (Baoji, Shanxi, China) and dissolved in sterile PBS. For *in vivo* administration, mice were intraperitoneally injected with 10 mg/kg Fan or the same volume of PBS once a day from 1 week before Aβ_1-42_ microinjection to the end of the behavioral tests. The behavioral tests were performed 2 weeks after Aβ_1-42_ microinjection.

### Morris water maze test

Morris water maze test is widely used to evaluate the spatial learning and memory ability of experimental animals (Wierwille et al., 2011; Othman et al., 2022). The test was performed in a circular stainless steel pool (150 cm in diameter, 50 cm in height) filled with water (22°C ± 1°C) and the water was made opaque with nontoxic white paint. The pool was surrounded by light blue curtains to form a relatively independent space, and three objects of different geometric shapes were pasted on the curtains as visual references. A high-definition camera was installed directly above the pool to record the animals’ movements. The test was divided into three stages: adaptation, training and probe test. During the adaptation phase, mice were put into the water and allowed to swim freely for 2 min to acclimate the maze. During the training phase, a hidden platform (7.5 cm in diameter) was equipped 1 cm below the water surface. The mice were trained for 5 consecutive days with 4 trials per day. In each trial, mice were put into the water and allowed to explore freely in the pool. If the mice found the hidden platform within 2 min and stayed there for 3 s, the trial was over. Otherwise, the mice were guided to the hidden platform after 2 min of exploration and required to stay there for 20 s. During the probe test when the hidden platform was removed, the mice were put into the water and allowed to explore freely for 2 min. All trials were recorded and analyzed by ANY-maze video tracking system (Stoelting, USA).

### Cell culture and treatment

N2A^APP^ cells, the mouse neuroblastoma N2A cells stably transfected with human Swedish mutant APP695, were generously provided by Professor Chunjiu Zhong (Fudan University, Shanghai, China). To create the mutant APP695 plasmid, we initiated the amplification of the wild-type DNA sequence of APP695 through PCR using human cDNA. This amplified sequence was subsequently inserted into a pcDNA 4.0 vector containing neomycin. Next, using gene mutation technology, we specifically introduced two mutations into the APP sequence to mimic the Swedish mutant APP695. We substituted the 595th lysine (K) with asparagine (N) and the 596th methionine (M) with leucine (L). This modified APP plasmid was then transfected into N2A cells. Through a stringent selection process involving the antibiotic G418 (Gibco, New York, USA), we eliminated N2A cells that did not successfully incorporate the mutant APP plasmid, while retaining those that did, resulting in the establishment of N2A^APP^ cells. The cells were cultured in medium composed of 90% Dulbecco’s modified Eagle’s medium (DMEM) (Gibco, New York, USA), 10% fetal bovine serum (FBS) (Gibco, New York, USA) and 50 μg/mL G418 and grown in a 37°C incubator with 5% CO_2_ humidified atmosphere.

To determine the optimal concentration of Fan in cells, N2A^APP^ cells were treated with gradient concentrations of Fan (0, 1.25, 2.5, 5, 10, and 20 μM) for 24 h.

For the BACE1 degradation experiment, N2A^APP^ cells were pretreated with 2.5 μM Fan or control solvent for 6 h followed by treatment with 100 μg/mL cycloheximide (CHX) (CST, Danvers, MA, United States) for different time (0, 3, 6 and 12 h) (Du et al., 2019).

To detect the degradation pathway of BACE1, N2A^APP^ cells were treated with 10 μM MG132 (MCE, Shanghai, China) or 50 μM chloroquine (CQ) (Sigma, St. Louis, MO, USA) for 24 h to block the ubiquitin-proteasome pathway or autophagy-lysosome pathway, respectively (Lai et al., 2019). In order to further verify whether Fan affects the ubiquitin-proteasome degradation or autophagy-lysosome degradation of BACE1, N2A^APP^ cells were pretreated with 10 μM MG132 or 50 μM CQ for 1 h followed by treatment with 2.5 μM Fan for 24 h.

### Cell viability assay

N2A^APP^ cells were plated onto 96-well plates and treated with gradient concentrations of Fan (0, 1.25, 2.5, 5, 10 and 20 μM) for 24 h when they reached 70%–80% confluence. Next, 10 μL Cell Counting Kit-8 (MCE, Shanghai, China) reagent was added to each well and the plates were incubated at 37°C for 3 h away from light. The cell viability was indicated by an absorbance at 450 nm, which was measured by a microplate reader (Thermo Fisher Scientific, MA, USA). For each group, four independent experiments run in triplicates were performed.

### Antibodies

Anti-C20 (1:1000) antibody used to detect APP and its β-CTFs was kindly provided by laboratory of Professor Weihong Song. Anti-BACE1 (1:1000, ab183612) and anti-PS1 (1:1000, ab76083) antibodies were purchased from Abcam (Cambridge, MA, USA). Anti-Ubquitin (1:1000, #10201-2-AP), anti-Beclin-1 (1:1000, #11306-1-AP), anti-Nrf2 (1:1000, #10396-1-AP), anti-HO-1(1:1000, #10701-1-AP), anti-SOD-1 (1:1000, #10269-1-AP), and anti-caspase-3 (1:1000, #66470-2-Ig) antibodies were obtained from Proteintech (Wuhan, Hubei, China). Anti-P62 (1:1000, H00008878-M01), anti-LC3 (1:1000, #12741) and anti-β-actin (1:3000, A5411) antibodies were purchased from Abnova (Taipei, Taiwan, China), Cell Signaling Technology (Danvers, MA, USA) and Sigma (St. Louis, MO, USA), respectively.

### Western blot

The cells or mouse brain tissues (cortex and hippocampus) were lysed on ice in RIPA lysis buffer (Beyotime, Shanghai, China) containing protease inhibitors (Roche, Basel, Switzerland) for 30 min. The lysates were then centrifuged at 12,000 rpm for 15 min at 4°C to collect the supernatant, which was the total protein that we need. After determination of protein concentration with a BCA Protein Assay Kit (Thermo Fisher Scientific, MA, USA), 30 µg of total protein was denatured by boiling with 5 × sample buffer at 95°C for 5 min. The samples were then separated by tris-glycine SDS-PAGE gels (EpiZyme, Shanghai, China) and transferred to immobilon-PTM polyvinylidene difluoride (PVDF) membranes (Millipore, MA, USA). To block the nonspecific binding, the membranes were incubated with 10% bovine serum albumin (Sigma, St. Louis, MO, USA) for 1.5 h at room temperature. Next, the membranes were incubated with primary antibodies overnight at 4°C followed by incubation with corresponding HRP-labeled goat anti-rabbit IgG (1: 3000, Perkin-Elmer) or goat anti-mouse IgG (1: 3000, Perkin-Elmer) for 1–2 h at room temperature. The membranes were finally imaged by the Bio-Rad Imager using Western ECL substrate (Bio-Rad, Hercules, CA, USA). The relative level of target protein was calculated using Quantity One software (Bio-Rad, Hercules, CA, USA), normalized to marker protein β-actin. For all Western blot analyses, there were at least 3 and at most 8 samples per group.

### Co-immunoprecipitation (Co-IP)

The cells were lysed on ice in cell lysis buffer for Western and IP (Beyotime, Shanghai, China) supplemented with protease inhibitors for 30 min, and then centrifuged at 12,000 g for 15 min at 4°C to collect the supernatants. After determination of protein concentration by using a BCA Protein Assay Kit, 500 µg of protein samples were incubated with anti-BACE1 primary antibody or nonspecific IgG as a control overnight at 4°C. Subsequently, the mixture was incubated with protein A/G magnetic beads for IP (Bimake, Shanghai, China) for additional 2 h at 4°C. Next, the beads were washed with ice-cold PBS for 4 times and the bound proteins on the beads were eluted by boiling in 1 × SDS-PAGE loading buffer at 95°C for 5 min, which were subjected to subsequent immunoblotting analysis.

### Quantitative real-time PCR (qRT-PCR)

Total RNA was extracted from cultured cells using High Pure Total RNA Extraction Kit (Bio Teke, Peking, China) according to the manufacturer’s instructions and the concentration and purity of RNA were determined using a spectrophotometer NanoDrop 2000 (Nanodrop Technologies, Wilmington, DE, USA). Next, 1 μg of total RNA was utilized as template to synthesize the corresponding single-stranded complementary DNA (cDNA) using the PrimeScript™ RT Reagent Kit (Takara, Otsu, Shiga, Japan). qRT-PCR of the cDNA was performed using SYBR Premix Ex Taq II (Takara, Otsu, Shiga, Japan) with CFX Manager software (Bio-Rad, Hercules, CA, USA). The primer sequences were as follows: BACE1 (forward: 5′- CAG​GGC​TAC​TAT​GTG​GAG​ATG​AC, reverse: 5′- GAG​TCA​AAG​AAG​GGC​TCC​AAA​GA); GAPDH (forward: 5′- GGC​ATT​GTG​GAA​GGG​CTC​AT, reverse: 5′- AGA​TCC​ACG​ACG​GAC​ACA​TT). GAPDH was used as an internal control for normalization, and the relative mRNA level of BACE1 were normalized to that of GAPDH.

### Oxidative stress analysis

The brain tissues (cortex and hippocampus) of mice were prepared into 10% homogenate in saline on ice, and then centrifuged at 2500 rpm for 15 min at 4°C to collect the supernatant. The levels of pro-oxidants including hydrogen peroxide (H_2_O_2_), total nitric oxide synthase (T-NOS) and inducible nitric oxide synthase (i-NOS) as well as antioxidants including glutathione reductase (GR) and total antioxidant capacity (T-AOC) in the above supernatant were measured by using related commercial kits (Jiancheng Biochemical, Nanjing, Jiangsu, China) as their instructions.

For the detection of H_2_O_2_, the supernatant was mixed with reagents 1 and 2 and incubated at 37°C for 1 min. Then, reagents 3 and 4 were added to the mixture and thoroughly mixed, and the absorbance at 405 nm was determined by a microplate reader.

For the detection of NOS, after mixing with (for i-NOS) or without (for T-NOS) reagent 6, the supernatant was incubated with reagents 1, 2 and 3 at 37°C for 15 min. Following this, reagents 4 and 5 were introduced to the mixture and thoroughly mixed. Afterward, a microplate reader was used to record the absorbance at 530 nm.

For the detection of GR, the supernatant was incubated with the working solution at 37°C for 30 s, and the absorbance A1 was recorded at 340 nm. After incubating at 37°C for an additional 10 min, the absorbance A2 was recorded at 340 nm. Ultimately, the GR level was presented as the difference between A1 and A2.

For the detection of T-AOC, the supernatant was mixed with reagents 1, 2 and 3 and incubated at 37°C for 30 min. Subsequently, reagents 4 and 5 were introduced to the mixture and incubated at 37°C for 10 min. Finally, the absorbance at 520 nm was recorded.

### Cholinesterase (AChE) activity assay

AChE activity was measured using an AChE assay kit (BC 2025, Solarbio, Peking, China) following the manufacturer’s instructions. In brief, mouse brain tissues (cortex and hippocampus) were homogenized to create an 8% homogenate in an extraction solution kept on ice. The homogenate was then subjected to centrifugation at 8000 *g* for 10 min at 4°C to obtain the supernatant. Subsequently, 15 µL of the supernatant was combined with 20 µL of reagent 2 and incubated at 37°C for 5 min. Following the addition of 50 µL of reagent 4, the mixture was centrifuged at 12,000 rpm for 5 min to collect the supernatant. Afterward, 10 µL of the supernatant, 170 µL of reagent 1, and 20 µL of reagent 3 were transferred to a 96-well plate. Following a 2-min incubation, the absorbance at 412 nm was recorded.

### Statistics

All statistical analyses were conducted with SPSS 22.0 software by using one-way ANOVA, repeated measures two-way ANOVA or two-tailed Student’s t-tests as appropriate. The results were presented as mean ± the standard error of the mean (SEM). The statistical significance was set as *p* < 0.05.

## Results

### Fan inhibits the amyloidogenic processing of APP by attenuating BACE1 expression in N2A^APP^ cells

**FIGURE 1:**
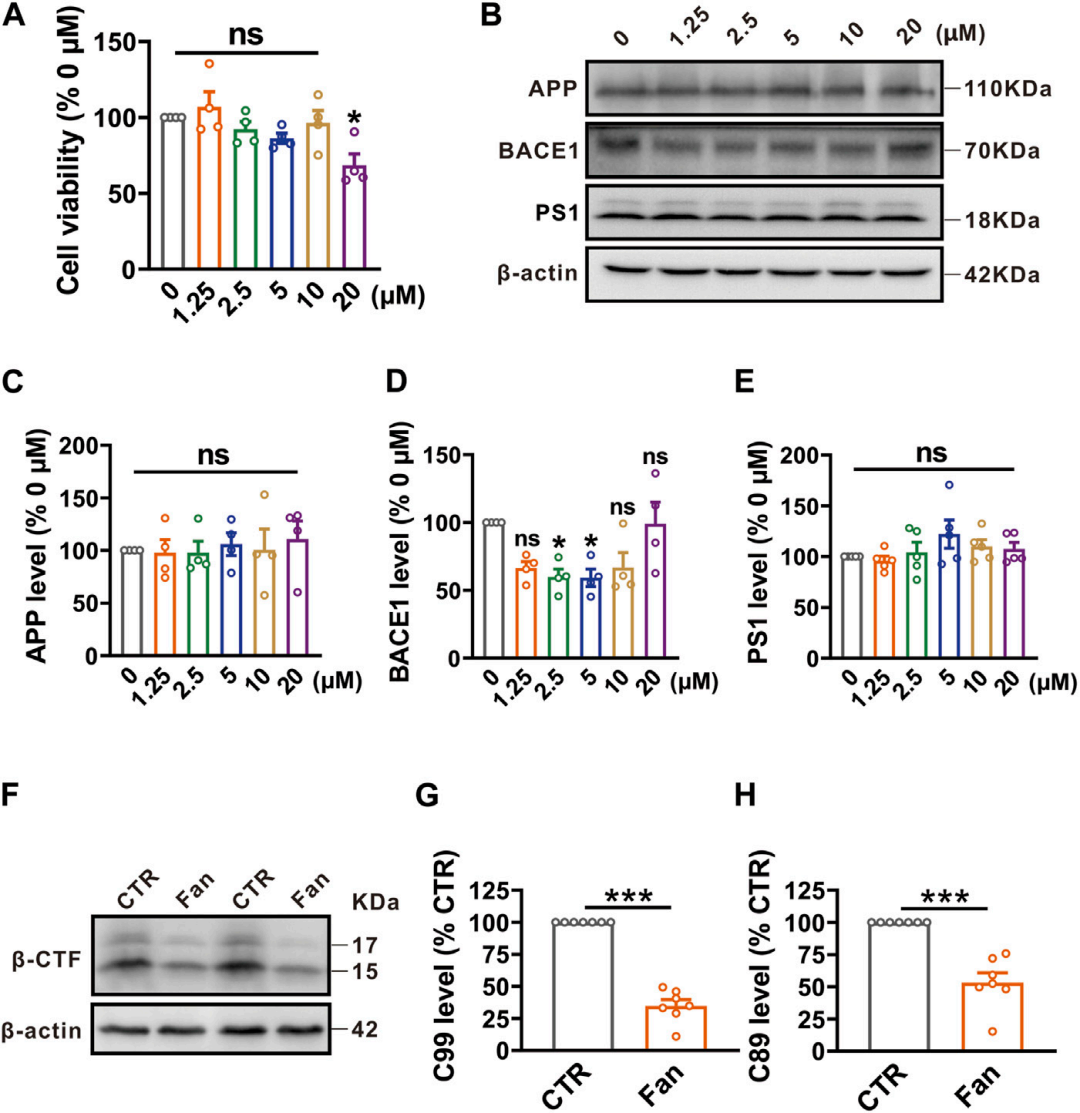
Fan inhibits APP amyloidogenic processing by targeting BACE1 in N2A^APP^ cells. **(A)** The cell viability assessed by CCK-8 in N2A^APP^ cells treated with gradient concentrations of Fan (0–20 μM) for 24 h. n = 4 in each group. **(B–E)** The protein levels of APP **(B,C)**, BACE1 **(B,D)** and PS1 **(B,E)** assessed by Western blot in N2A^APP^ cells treated with gradient concentrations of Fan (0–20 μM) for 24 h. n = 4–5 in each group. **(F–H)** The protein levels of C99 **(F,G)** and C89 **(F,H)** assessed by Western blot in N2A^APP^ cells treated with 2.5 μM of Fan for 24 h. n = 7 in each group. Data are presented as mean ± SEM, *p < 0.05, ***p < 0.001.

To assess the effect of Fan on cell survival, N2A^APP^ cells were treated with gradient concentrations (ranging from 0 to 20 μM) of Fan for 24 h, and then cell viability was determined by using the CCK-8 assay. All concentrations of Fan had no effect on the cell viability of N2A^APP^ cells, apart from the 20 μM concentration which exhibited a reduction in cell viability (1.25 μM: 106.95% ± 10.01%, *p* = 0.973 vs. 0 μM; 2.5 μM: 92.27% ± 5.09%, *p* = 0.958 vs. 0 μM; 5 μM: 86.33% ± 3.36%, *p* = 0.686 vs. 0 μM; 10 μM: 96.39% ± 8.20%, *p* = 0.999 vs. 0 μM; 20 μM: 68.61% ± 7.40%, *p* = 0.034 vs. 0 μM; n = 4 in each group; Figure 1A). Give the crucial role of Aβ in the AD pathogenesis, we therefore test the effect of Fan on APP amyloidogenic processing in N2A^APP^ cells. N2A^APP^ cells were treated with the aforementioned concentrations (ranging from 0 to 20 μM) of Fan for 24 h. It was observed that Fan treatment at concentrations of 2.5 μM and 5 μM significantly reduced the protein levels of BACE1 (1.25 μM: 66.38% ± 4.74%, *p* = 0.130 vs. 0 μM; 2.5 μM: 59.83% ± 5.84%, *p* = 0.049 vs. 0 μM; 5 μM: 59.18% ± 6.38%, *p* = 0.044 vs. 0 μM; 10 μM: 66.77% ± 10.95%, *p* = 0.138 vs. 0 μM; 20 μM: 99.05% ± 16.01%, *p* = 1.000 vs. 0 μM; n = 4 in each group; Figure 1D). However, all concentrations of Fan had no obvious effect on the expression of APP (1.25 μM: 97.79% ± 12.59%, *p* = 1.000 vs. 0 μM; 2.5 μM: 97.81% ± 10.96%, *p* = 1.000 vs. 0 μM; 5 μM: 105.97% ± 10.89%, *p* = 1.000 vs. 0 μM; 10 μM: 100.58% ± 19.70%, *p* = 1.000 vs. 0 μM; 20 μM: 110.86% ± 17.13%, *p* = 0.992 vs. 0 μM; n = 4 in each group; Figure 1C) and PS1 (1.25 μM: 96.04% ± 4.36%, *p* = 0.999 vs. 0 μM; 2.5 μM: 104.25% ± 9.79%, *p* = 0.999 vs. 0 μM; 5 μM: 122.17% ± 13.83%, *p* = 0.402 vs. 0 μM; 10 μM: 109.94% ± 6.61%, *p* = 0.950 vs. 0 μM; 20 μM: 107.54% ± 6.40%, *p* = 0.985 vs. 0 μM; n = 5 in each group; Figure 1E). Furthermore, β-CTFs, the direct byproducts of APP cleavage by BACE1 were detected in N2A^APP^ cells treated with a concentration of 2.5 μM of Fan for 24 h. A dramatic decrease in the protein levels of both C99 (n = 7, 34.83% ± 4.82%, *p* < 0.001 vs. 0 μM; Figure 1G) and C89 (n = 7, 53.28% ± 7.52%, *p* < 0.001 vs. 0 μM; Figure 1H) was observed with Fan treatment. These data indicate that Fan alleviates APP amyloidogenic processing by inhibiting BACE1 expression.

### Fan promotes autophagy-lysosomal degradation of BACE1

**FIGURE 2:**
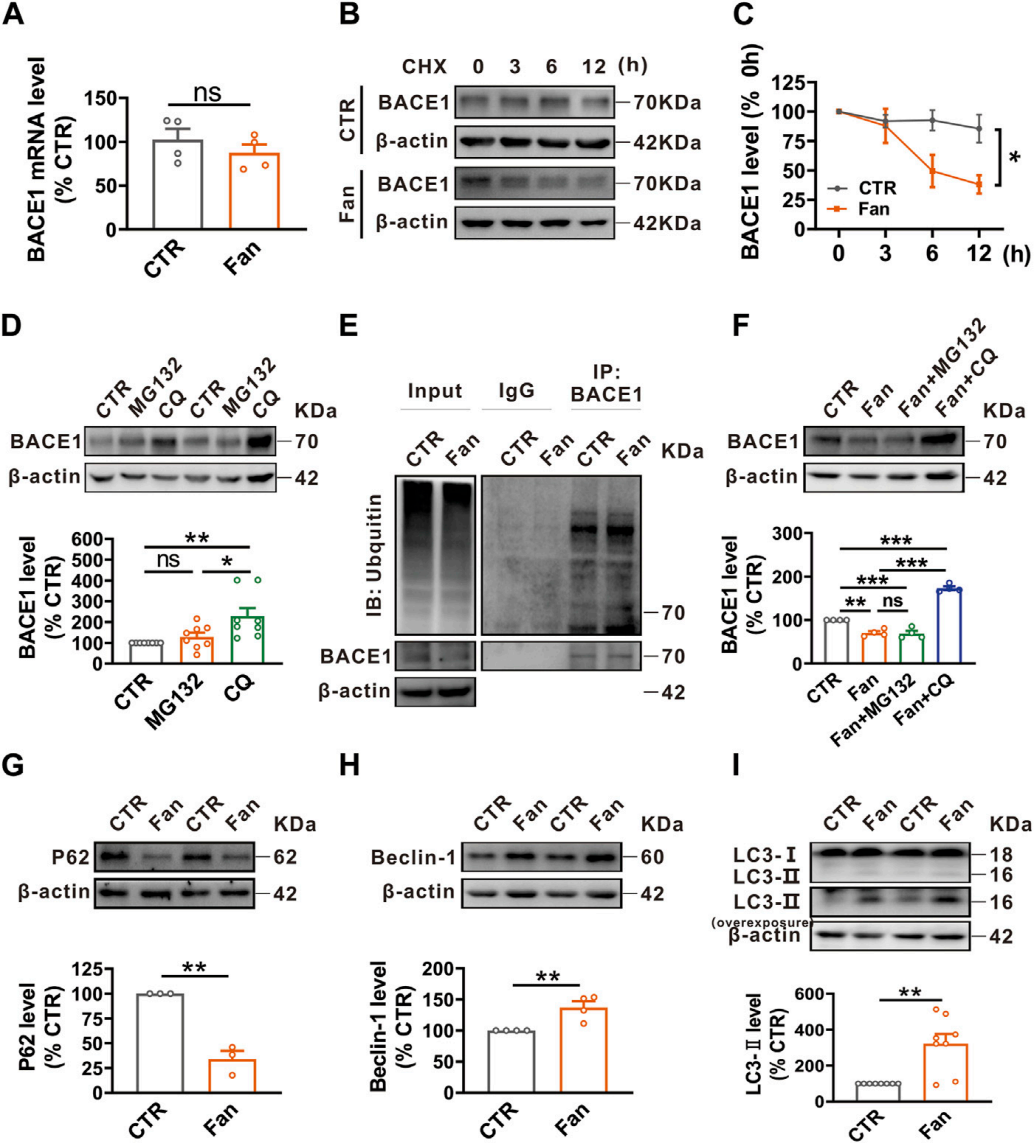
Fan promotes autophagy-lysosomal degradation of BACE1. **(A)** The mRNA level of BACE1 assessed by qRT-PCR in N2A^APP^ cells treated with Fan (2.5 μM for 24 h). n = 4 in each group. **(B,C)** The effect of Fan on the degradation of BACE1 assessed by half-life measurements in N2A^APP^ cells treated with 100 μg/mL cycloheximide (CHX). n = 5–6 in each group. **(D)** The protein level of BACE1 assessed by Western blot in N2A^APP^ cells treated with proteasome inhibitor MG132 (10 μM for 24 h) or lysosome inhibitor chloroquine (CQ, 50 μM for 24 h). n = 8 in each group. **(E)** The total ubiquitination level and the ubiquitination level of BACE1 assessed by Western blot and Co-IP in N2A^APP^ cells treated with Fan (2.5 μM for 24 h). n = 2 in each group. **(F)** The protein level of BACE1 assessed by Western blot in N2A^APP^ cells treated with Fan (2.5 μM for 24 h) along with or without 1 h of pretreatment with MG132 (10 μM for 25 h) or CQ (50 μM for 25 h). n = 4 in each group. **(G–I)** The protein levels of P62 **(G)**, Beclin-1 **(H)** and LC3 **(I)** assessed by Western blot in N2A^APP^ cells treated with Fan (2.5 μM for 24 h). n = 3–8 in each group. Data are presented as mean ± SEM, *p < 0.05, **p < 0.01, ***p < 0.001.

The protein homeostasis is determined by the protein synthesis and degradation, therefore we examined the influence of Fan on the synthesis and degradation of BACE1. To elucidate the role of Fan in BACE1 synthesis, qRT-PCR was employed to detect the BACE1 mRNA level in N2A^APP^ cells treated with Fan (2.5 μM for 24 h). Fan treatment was observed to have no effect on the BACE1 mRNA level (n = 4, 87.30% ± 9.62%, *p* = 0.376 vs. 0 μM; Figure 2A). To test the effect of Fan on the degradation of BACE1, a protein synthesis inhibitor, cycloheximide (CHX), was introduced to N2A^APP^ cells in the presence or absence of Fan treatment to inhibit protein synthesis. The results elucidated that Fan-treated N2A^APP^ cells accelerated the degradation of BACE1 compared with those without Fan treatment (*p* = 0.035 vs. CTR; n = 5–6 in each group; Figures 2B, C). The ubiquitin-proteasome system and the autophagy-lysosome system are two major protein degradation pathways in eukaryotic cells (Sun-Wang et al., 2020). To further determine the degradation pathway of BACE1, N2A^APP^ cells were treated with either proteasome inhibitor MG132 (10 μM for 24 h) or lysosomal inhibitor chloroquine (CQ, 50 μM for 24 h) and the protein level of BACE1 was subsequently detected. It was found that CQ (n = 8, 228.14% ± 39.30%, *p* = 0.005 vs. CTR; Figure 2D), rather than MG132 (n = 8, 128.78% ± 19.95%, *p* = 0.707 vs. CTR; Figure 2D), led an apparent increase in the protein level of BACE1, indicating that BACE1 degradation primarily transpires through the autophagy-lysosome system. Meanwhile, we examined the effects of Fan on ubiquitination in N2A^APP^ cells treated with Fan and found that Fan did not exert an impact on the total ubiquitination level or on the ubiquitination level of BACE1 (n = 2 in each group; Figure 2E). Furthermore, we detected the protein level of BACE1 in N2A^APP^ cells treated with Fan for 24 h with or without 1 h of pretreatment with MG132 or CQ. The results showed that CQ, but not MG132, counteracted the inhibitory effect of Fan on BACE1 protein levels (Fan: 70.34% ± 3.93%, *p* = 0.002 vs. CTR; Fan + MG132: 68.92% ± 6.01%, *p* = 0.995 vs. Fan; Fan + CQ: 173.2% ± 4.26%, *p* < 0.001 vs. Fan; n = 4 in each group; Figure 2F). Simultaneously, Fan was found to augment autophagy, as evidenced by the decrease in P62 levels (n = 3, 34.01% ± 8.38%, *p* = 0.001 vs. CTR; Figure 2G) and the increase in Beclin-1 (n = 4, 137.27% ± 9.80%, *p* = 0.009 vs. CTR; Figure 2H) and LC3-II (n = 8, 321.48% ± 54.22%, *p* = 0.001 vs. CTR; Figure 2I) levels. Furthermore, we detected the effect of Fan treatment on BACE1 SUMOylation, and the results demonstrated that Fan treatment had no effect on the SUMOylation of BACE1 in N2A^APP^ cells (n = 4 in each group; Supplementary Figure S1). Taken together, these results suggest that Fan facilitates autophagy-lysosomal degradation of BACE1.

### Fan rescues cognitive impairment in Aβ_1-42_-induced mouse model of AD

**FIGURE 3:**
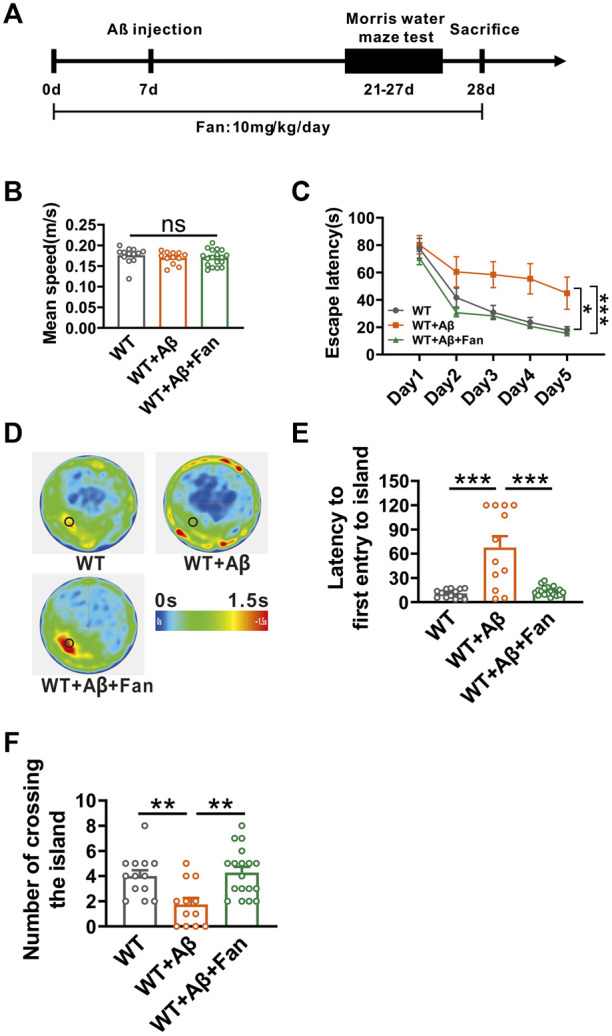
Fan attenuates Aβ_1-42_-induced spatial learning and memory deficits in mice. **(A)** The flow chart of animal experiment. **(B)** Mean speed travelled in the Morris water maze during adaptation phase. n = 12–18 in each group. **(C)** The escape latency for finding the hidden platform during training sessions in the Morris water maze. n = 12–18 in each group. **(D)** Average heatmap during the probe test in the Morris water maze. **(E)** The latency to first entry to the platform-zone during the probe test in the Morris water maze. n = 12–18 in each group. **(F)** The number of entries into the platform-zone during the probe test in the Morris water maze. n = 12–18 in each group. Data are presented as mean ± SEM, *p < 0.05, **p < 0.01, ***p < 0.001.

Given that Fan inhibits APP amyloidogenic processing, we therefore tested the effect of fan on the cognitive function of AD mice. We established a mouse model of AD via the microinjection of Aβ_1-42_ into lateral ventricle of WT mice. The therapeutic efficacy of Fan was subsequently tested by intraperitoneal administration of 10 mg/kg Fan or an equivalent volume of PBS once a day, starting 1 week prior to Aβ_1-42_ microinjection and continued until the culmination of the behavioral tests (Figure 3A). Two weeks after Aβ_1-42_ administration, mice were subjected to Morris water maze test to evaluate the spatial learning and memory abilities (Figure 3A). During the adaptation period, there was no difference in the average swimming speed travelled in the water maze among three groups (WT: 0.176 ± 0.006 m/s; WT + Aβ: 0.170 ± 0.004 m/s, *p* = 0.672 vs. WT; WT + Aβ+Fan: 0.170 ± 0.005 m/s, *p* = 1.000 vs. WT + Aβ, *p* = 0.624 vs. WT; n = 12–18 in each group; Figure 3B), suggesting that administration of Aβ_1-42_ or Fan had no appreciable influence on the motor function of mice. During the training phase, Aβ_1-42_ treated mice exhibited significant deficits in spatial learning, as evidenced by the prolonged escape latency to find the hidden platform compared with the WT mice (*p* = 0.014 vs. WT; n = 12–18 in each group; Figure 3C). However, Fan treatment dramatically shortened the escape latency in Aβ_1-42_ treated mice (*p* < 0.001 vs. WT + Aβ; *p* = 0.716 vs. WT; n = 12–18 in each group; Figure 3C). In the probe test, Aβ_1-42_ treated mice exhibited impaired spatial memory retrieval. This was reflected in a prolonged latency to first entry into platform-zone (WT: 10.74 ± 1.46 s; WT + Aβ: 67.63 ± 13.97 s, *p* < 0.001 vs. WT; n = 12–18 in each group; Figure 3E) and a reduction in the number of platform-zone crossing (WT: 4 ± 0.47; WT + Aβ: 1.75 ± 0.51, *p* = 0.009 vs. WT; n = 12–18 in each group; Figure 3F), which were restored to normal conditions by Fan treatment (for first entry into platform-zone: WT + Aβ+Fan: 13.66 ± 1.35 s, *p* < 0.001 vs. WT + Aβ, *p* = 0.948 vs. WT, n = 12–18 in each group, Figure 3E; for platform-zone crossing: WT + Aβ+Fan: 4.28 ± 0.45, *p* = 0.001 vs. WT + Aβ, *p* = 0.906 vs. WT, n = 12–18 in each group; Figure 3F). Taken together, these results demonstrate that Fan ameliorates cognitive decline in Aβ_1-42_-induced mouse model of AD.

### Fan rescues autophagic function in Aβ_1-42_-induced mouse model of AD

**FIGURE 4:**
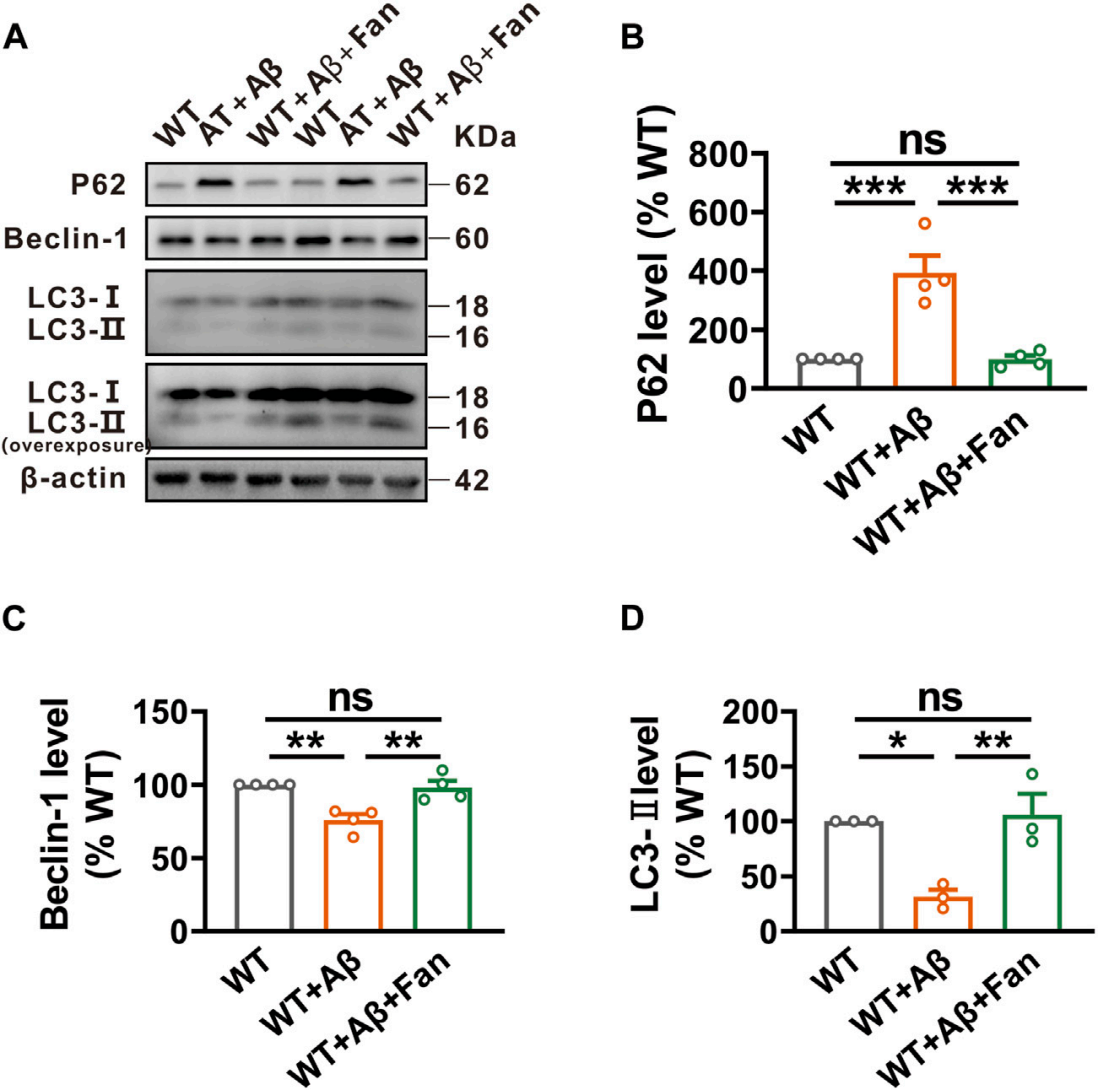
Fan induces autophagy in Aβ_1-42_-treated mice. **(A–D)** The protein levels of P62 **(A,B)**, Beclin-1 **(A,C)** and LC3 **(A,D)** assessed by Western blot in the brain tissues of mice treated with Aβ_1-42_ together with or without Fan. n = 3–4 in each group. Data are presented as mean ± SEM, *p < 0.05, **p < 0.01, ***p < 0.001.

Give Fan’s capacity to promote autophagy in N2A^APP^ cells, an *in vitro* AD model, we undertook to investigate Fan’s potential role on regulating autophagy in the Aβ_1-42_-induced mouse model of AD. It was found that Aβ_1-42_ treated mice showed obviously impaired autophagic function compared with WT mice, as demonstrated by the increased levels of P62 (n = 4, 392.88% ± 58.52%, *p* < 0.001 vs. WT; Figure 4B) and the decreased levels of Beclin-1 (n = 4, 75.87% ± 4.02%, *p* = 0.002 vs. WT; Figure 4C) and LC3-II (n = 3, 31.58% ± 6.36%, *p* = 0.013 vs. WT; Figure 4D). Remarkably mirroring the *in vitro* results, Fan treatment was found to facilitate autophagy in Aβ_1-42_-induced mouse model of AD, as shown by the restoration of P62 (n = 4, 99.40% ± 13.12%, *p* < 0.001 vs. WT + Aβ, *p* = 1.000 vs. WT; Figure 4B), Beclin-1 (n = 4, 98.08% ± 4.54%, *p* = 0.004 vs. WT + Aβ, *p* = 0.922 vs. WT; Figure 4C) and LC3-II (n = 3, 106.14% ± 18.81%, *p* = 0.009 vs. WT + Aβ, *p* = 0.925 vs. WT; Figure 4D).

### Fan rescues oxidative stress and apoptosis in Aβ_1-42_-induced mouse model of AD

**FIGURE 5:**
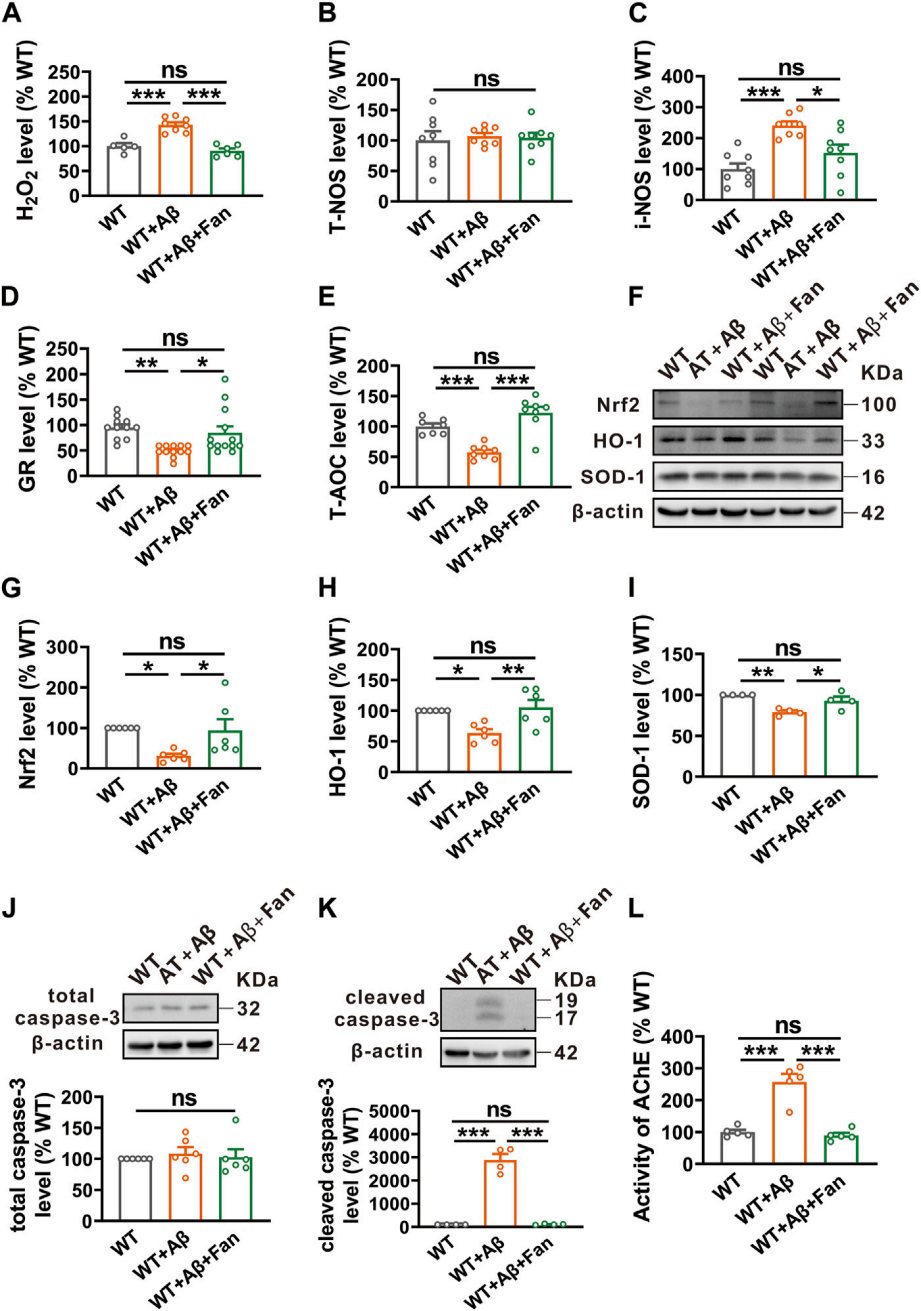
Fan inhibits Aβ_1-42_-induced oxidative stress and apoptosis in mice. **(A–C)** The levels of pro-oxidants H_2_O_2_ **(A)**, T-NOS **(B)** and i-NOS **(C)** assessed by using related commercial kits in the brains of mice treated with Aβ_1-42_ together with or without Fan. n = 5–8 in each group. **(D,E)** The levels of antioxidants GR **(D)** and T-AOC **(E)** assessed by using related commercial kits in the brains of mice treated with Aβ_1-42_ together with or without Fan. n = 7–13 in each group. **(F–I)** The protein levels of antioxidant factors Nrf2 **(F,G)**, HO-1 **(F,H)** and SOD-1 **(F,I)** assessed by Western blot in the brain tissues of mice treated with Aβ_1-42_ together with or without Fan. n = 4–6 in each group. **(J,K)** The protein levels of total caspase-3 **(J)** and cleaved caspase-3 **(K)** assessed by Western blot in the brain tissues of mice treated with Aβ_1-42_ together with or without Fan. n = 4–6 in each group. **(L)** The AChE activity assessed by using an AChE assay kit in the brains of mice treated with Aβ_1-42_ together with or without Fan. n = 5 in each group. Data are presented as mean ± SEM, *p < 0.05, **p < 0.01, ***p < 0.001.

Oxidative stress is a downstream pathological consequence of Aβ pathology and plays an essential role in the pathogenesis of AD (Ochi and DeGroot, 1969). Thus, the potential role of Fan in oxidative stress was explored in Aβ_1-42_-induced mouse model of AD. By using related commercial kits, we found elevated levels of pro-oxidants including hydrogen peroxide (H_2_O_2_) (143.00% ± 5.17%, *p* < 0.001 vs. WT; n = 5–8 in each group; Figure 5A) and inducible nitric oxide synthase (i-NOS) (241.04% ± 12.56%, *p* < 0.001 vs. WT; n = 8 in each group; Figure 5C) in the Aβ_1-42_ treated mice. Conversely, the levels of antioxidants such as glutathione reductase (GR) (52.08% ± 3.38%, *p* = 0.003 vs. WT; n = 11–13 in each group; Figure 5D) and total antioxidant capacity (T-AOC) (57.49% ± 4.06%, *p* < 0.001 vs. WT; n = 7–8 in each group; Figure 5E) were apparently decreased in Aβ_1-42_ treated mice. Notably, Fan treatment rescued both the increased pro-oxidants and the decreased antioxidants to normal levels (for H_2_O_2_: 90.67% ± 4.83%, *p* < 0.001 vs. WT + Aβ, *p* = 0.504 vs. WT, n = 5–8 in each group, Figure 5A; for i-NOS: 152.60% ± 26.39%, *p* = 0.013 vs. WT + Aβ, *p* = 0.171 vs. WT, n = 8 in each group; Figure 5C; for GR: 89.42% ± 13.19%, *p* = 0.017 vs. WT + Aβ, *p* = 0.701 vs. WT, n = 11–13 in each group; Figure 5D; for T-AOC: 122.58% ± 9.74%, *p* < 0.001 vs. WT + Aβ, *p* = 0.080 vs. WT, n = 7–8 in each group; Figure 5E). Whereas, treatment with Aβ_1-42_ or Fan did not affect the level of total nitric oxide synthase (T-NOS) (WT + Aβ: 107.03% ± 5.15%, *p* = 0.882 vs. WT; WT + Aβ+Fan: 104.32% ± 8.29%, *p* = 0.982 vs. WT + Aβ, *p* = 0.953 vs. WT; n = 8 in each group; Figure 5B). It was further substantiated by Western blot. The protein levels of antioxidant factors including nuclear factor erythroid-2-related factor 2 (Nrf2) (n = 6, 30.90% ± 5.41%, *p* = 0.024 vs. WT; Figure 5G), heme oxygenase-1 (HO-1) (n = 6, 63.53% ± 6.31%, *p* = 0.014 vs. WT; Figure 5H) and superoxide dismutase-1 (SOD-1) (n = 4, 78.99% ± 2.10%, *p* = 0.003 vs. WT; Figure 5I) were significantly reduced in Aβ_1-42_ treated mice compared with WT mice, which were restored by Fan treatment (for Nrf2: n = 6, 93.91% ± 27.82%, *p* = 0.039 vs. WT + Aβ, *p* = 0.963 vs. WT, Figure 5G; for HO-1: n = 6, 105.41% ± 12.13%, *p* = 0.005 vs. WT + Aβ, *p* = 0.879 vs. WT; Figure 5H; for SOD-1: n = 4, 92.72% ± 5.15%, *p* = 0.035 vs. WT + Aβ, *p* = 0.294 vs. WT; Figure 5I). In addition, evaluation of apoptosis through the examination of caspase-3 protein levels demonstrated that the protein level of cleaved caspase-3 was obviously increased in Aβ_1-42_ treated mice compared with WT mice (n = 4, 2876.87% ± 267.61%, *p* < 0.001 vs. WT; Figure 5K), while Fan treatment countered this increase (n = 4, 108.59% ± 11.70%, *p* < 0.001 vs. WT + Aβ, *p* = 0.999 vs. WT; Figure 5K). Meanwhile, there was no difference in total caspase-3 among the three groups (WT + Aβ: 108.37% ± 10.52%, *p* = 0.806 vs. WT; WT + Aβ+Fan: 102.86% ± 12.39%, *p* = 0.910 vs. WT + Aβ, *p* = 0.975 vs. WT; n = 6 in each group; Figure 5J). Furthermore, we determined the effect of Fan on AChE activity in the brain tissues of Aβ_1-42_-induced mouse model of AD. It was found that the AChE activity was apparently increased in Aβ_1-42_ treated mice compared with WT mice (n = 5, 257.42% ± 25.07%, *p* < 0.001 vs. WT; Figure 5L), which was restored to normal levels with Fan administration (n = 5, 89.80% ± 7.76%, *p* < 0.001 vs. WT + Aβ, *p* = 0.891 vs. WT; Figure 5L). These results indicate that Fan rescues Aβ_1-42_-induced oxidative stress and apoptosis in AD model mice.

## Discussion

In the current study, we demonstrate that Fan treatment alleviates the amyloidogenic processing of APP by promoting autophagy-lysosomal degradation of BACE1. Furthermore, our study indicates that Fan treatment diminishes oxidative stress and the consequential mitigation of oxidative stress-induced apoptosis, leading to improvement of cognitive dysfunction in Aβ_1-42_-induced mouse model of AD. Collectively, our present study confirms the neuroprotective effects of Fan on AD, indicating Fan may be a potential therapeutic agent for AD.

A substantial body of evidence has indicated the deposition of Aβ as a principal etiological factor in the development of AD (Hardy and Allsop, 1991; Hardy and Higgins, 1992; Selkoe and Hardy, 2016), and BACE1 serves as a critical rate-limiting enzyme in the biosynthetic pathway that culminates in the formation of Aβ peptides. Existing research has elucidated two principal degradation pathways of BACE1: the ubiquitin-proteasome pathway and the autophagy-lysosome pathway (Sun-Wang et al., 2020). Notably, proteasome inhibitors such as MG132 have been observed to lead to upregulation of BACE1 expression in 2EB2 cells that co-express the Swedish mutant APP and BACE1 (Qing et al., 2004; Zhang et al., 2012). Contrary to these findings, our study employing N2A cells with APP-only overexpression revealed that MG132 treatment failed to exert a significant effect on the BACE1 degradation. This discrepancy in outcomes could potentially be attributed to the phenomenon of BACE1 overexpression in 2EB2 cells. Simultaneously, lysosomal inhibitors including CQ are reported to lead to an increase in both ectopically expressed BACE1 and endogenous BACE1, alongside an accumulation of BACE1 in late endosomal or lysosomal compartments (Koh et al., 2005). Through concerted endeavors, it was ascertained that BACE1 is tagged by Lys-K63 ubiquitin chains, directing it to degradation via the autophagy pathway (Kang et al., 2010). Consistent with these findings, our results revealed that CQ, through inhibiting autophagy exerts a potent inhibitory effect on BACE1 degradation (Figure 2).

Autophagy is an essential cellular process that helps maintain cellular homeostasis by degrading and recycling damaged or unnecessary cellular components. The mammalian target of rapamycin (mTOR) signaling serves as a central regulator of autophagy (Glick et al., 2010). Extensive evidence has shown that signaling pathways that negatively regulate mTOR, such as the AMP-activated protein kinase (AMPK) signaling pathway, promote autophagy (Din et al., 2012). Conversely, signaling pathways that activate mTOR, including the phosphoinositide 3-kinase/protein kinase B (PI3K/Akt) and mitogen-activated protein kinase (MAPK) pathways, effectively inhibit autophagy process (Xue et al., 2017; Kinsey et al., 2019). However, numerous lines of evidence have revealed that Fan induces autophagy via the activation of AMPK/mTOR/ULK1 signaling pathway (Xiang et al., 2021). Meanwhile, Fan has also been reported to inhibit the PI3K/Akt and MAPK signaling pathways in several diseases (Li et al., 2017; Jiang F. et al., 2020; Chen et al., 2020; Villa et al., 2020; Chen et al., 2022). However, the exploration of Fan’s impact on proteasome regulation remains relatively limited and restricted to human cancer cells and articular chondrocytes (Chuu et al., 2015). In the current study, we found that Fan exerted a noticeable influence on autophagic function, subsequently facilitating lysosomal degradation of BACE1 and ultimately mitigating the Aβ generation (Figures 1, 2, 4). Nevertheless, it is noteworthy that Fan did not lead to significant changes in the total ubiquitination level and the specific BACE1 ubiquitination level in AD models (Figure 2).

Oxidative stress has been considered to be an essential central factor in the pathogenesis of AD due to its crucial role in the Aβ accumulation. In the AD brains, a large number of positive ions attached to the Aβ′s hydrophilic N terminus, undergoing redox reactions and generating substantial levels of ROS, leading to excessive release of ROS and inducing oxidative stress. In turn, this high level of oxidative stress perpetuates the feedback loop by increasing Aβ generation, thereby establishing a vicious cycle and exacerbating AD progression (Kim et al., 2015; Liu et al., 2017). Therefore, inhibition of oxidative stress might be an effective therapeutic strategy for AD.

Fan has drawn attention for its antioxidant and neuroprotective properties across diverse disease models. Notably, Fan has been demonstrated to inhibit cyanide and H_2_O_2_-induced neuronal cell death through the regulation of Ca^2+^ influx, inhibition of glutamate release and reduction of oxidants and ROS generation in cultured rat cerebellar granule cells (Cho and Seong, 2002; Koh et al., 2003). In addition, Fan shows a significant enhancement in SOD activity with a downregulation of Kelch-like ECH-associated protein 1 (Keap1). This modulation induces the activation of nuclear factor erythroid-2-related factor 2 (Nrf2), leading to a subsequent increase in the expression of antioxidant protein heme oxygenase-1 (HO-1), thus protecting HT22 cells from glutamate-induced oxidative damage (Bao et al., 2019). Furthermore, Fan ameliorates the neuronal injury induced by cerebral ischemia in neonatal rats, as evidenced by the restoration of inflammation and oxidative stress (Daicheng et al., 2018). In alignment with these findings, we found increased levels of oxidative stress characterized by diminished antioxidant expression, elevated pro-oxidant expression and an increase in apoptosis in Aβ_1-42_-induced mouse model of AD. More importantly, these effects can be significantly mitigated by Fan treatment (Figure 5).

Impairment of the cholinergic system has been noted to be a significant pathological feature of AD, and the inhibition of AChE activity serves as the foremost clinical approach to AD treatment (Galimberti and Scarpini, 2016). Meanwhile, prior research has indicated the robust AChE inhibitory properties of Fan (Kong et al., 2019; Kong et al., 2020). Consistent with these findings, our study revealed an increase in AChE activity in Aβ_1-42_-induced mouse model of AD, and notably, this elevated activity was fully restored through the administration of Fan (Figure 5).

AD is a chronic inflammatory disease of the central nervous system, and targeting neuroinflammation represents a promising approach for both the prevention and treatment of AD (Dhapola et al., 2021). Fan has been demonstrated to possess significant anti-inflammatory properties in various inflammatory diseases. Notably, Fan has been shown to exert anti-arthritic effects via the inhibition of MAPK pathway and NF-κB pathway in cell and animal models of rheumatoid arthritis (Villa et al., 2020). Meanwhile, Fan has exhibited the ability to attenuate LPS-induced endotoxemia by inhibiting ERK1/2 and NF-κB p65 phosphorylation (Chen et al., 2020). Furthermore, Fan has demonstrated efficacy in alleviating renal inflammation in a rat model of diabetic nephropathy by suppressing the P38 MAPK pathway (Jiang Y. et al., 2020). Further studies are needed to explore whether the anti-inflammatory effect of Fan and its related signaling pathway are involved in Fan-mediated improvement of cognitive function in AD.

Nonetheless, there is no direct evidence that Fan ameliorates the cognitive decline. Given its potent antioxidant properties and its capacity to inhibit Aβ generation, it is reasonable to speculate that Fan treatment may ameliorate Aβ_1-42_-induced cognitive dysfunction in mice (Figure 3).

In conclusion, our results demonstrate that Fan improves cognitive impairments via promoting autophagy and inhibiting oxidative stress, suggesting that Fan may be a potential therapeutic agent for AD.

## Data Availability

The original contributions presented in the study are included in the article/Supplementary Material, further inquiries can be directed to the corresponding authors.
