# Supplementary material for: Fangchinoline alleviates cognitive impairments through enhancing autophagy and mitigating oxidative stress in Alzheimer’s disease models
Source: Front Cell Dev Biol. 2023 Dec 11;11:1288506. doi: 10.3389/fcell.2023.1288506 (PMC10749363; doi:10.3389/fcell.2023.1288506)
Supplement: Supplementary file 1 [file Presentation1.pdf]

Supplementary Materials

Supplementary Figures

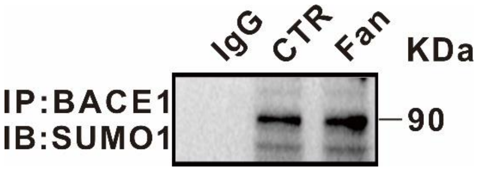

Supplementary Figure. S1. Fan had no effect on the SUMOylation of BACE1. The SUMOylation level of BACE1 assessed by Western blot and Co-IP in N2A^{APP} cells treated with Fan (2.5 μM for 24 h). n = 4 in each group.
